# Evaluation of the usefulness of a paper–pencil group cognitive assessment for older adults in the community

**DOI:** 10.1186/s12889-023-16119-3

**Published:** 2023-06-30

**Authors:** Daisuke Cho, Hiroyuki Suzuki, Susumu Ogawa, Tomoya Takahashi, Kenichiro Sato, Ai Iizuka, Momoko Kobayashi, Misako Yamauchi, Anna Kinai, Yan Li, Yoshinori Fujiwara

**Affiliations:** Research Team for Social Participation and Healthy Aging, Tokyo Metropolitan Institute for Geriatrics and Gerontology, 8F, Itabashi Center Building, 3-9-7, Itabashi, Itabashi-Ku, Tokyo, 173-0004 Japan

**Keywords:** Dementia, Cognitive decline, Paper–pencil type group examination for cognitive assessment

## Abstract

**Background:**

As the older population increases, the need for early detection of cognitive decline is also increasing. In this study, we examined whether our paper–pencil type group examination for cognitive assessment (PAPLICA) could detect the effects of years of education and aging.

**Methods:**

PAPLICA was conducted on 829 older people. The inclusion criteria were age 60 years or older and the ability to come to the event site alone. The exclusion criteria were participants with a medical or psychiatric disorder or dementia.One examiner conducted the test on a group of approximately 10–20 people in approximately 25 min. Participants were instructed on tackling the issues projected on the projector, and their answers were recorded in a response booklet.

**Results:**

An independent sample *t*-test was performed for years of education, and ANCOVA was performed for aging. Among the test items included in PAPLICA, the Speed I and Letter fluency tests were unable to detect the effects of aging. Furthermore, the age at which the effect of aging manifests varies depending on the test item. For instance, a decline in scores in the Speed I and Picture ECR Free recall tests was observed in the 70–74 age group; for that of Word DRT, Picture ECR cued recall, and Similarity, in the 75–79 age group; for CFT, in the 80–84 age group, and for CLOX, the decline was observed in the 85 ≤ age group.

**Conclusions:**

PAPLICA, similar to other neuropsychological tests, was able to detect the effects of years of education and aging. Future testing should be conducted on different demographics to identify the differences in patterns of cognitive decline.

## Background

The increasing prevalence of dementia is cited as a problem in an aging society. The number of reported cases of dementia is increasing annually, and the financial costs are enormous [1]. Japan is also facing simillar problem, and according to a survey in 2012. the prevalence of dementia was 15%. this rate continue incresing, reaching approximately 20% by 2025 [2]. To date, one way to prevent cognitive decline is through intellectual activity and novel learning, which is based on the cognitive reserve hypothesis [3]. Intellectual activities, such as taking pictures, playing Go, and reading aloud picture books, can help maintain and improve cognitive function [4–7]. Furthermore, even in mild cognitive impairment (MCI), such activities can improve cognitive function, [8] and a certain number of patients return to normal cognitive status [9]. Thus, the development of methods for early detection of cognitive decline is an important issue.

To assess cognitive function, examiners can use neuropsychological tests, such as the Mini-Mental State Examination (MMSE) [10], Montreal Cognitive Assessment (MoCA) [11], Wechsler Memory Scale-Revised, and Wechsler Adult Intelligence Scale-IV. These tests are conducted face-to-face between the examiner and the participant. However, considering the increasing older adults population and the importance of early detection of cognitive decline, cognitive assessments other than face-to-face neuropsychological testing are needed [12].

In preparation for the forthcoming situation, Suzuki (2010–2011) developed a new neuropsychological test that assesses a larger number of older adults in a shorter period of time, called the paper-pencil type group examination for cognitive assessment (PAPLICA). This neurocognitive test can be used in large-scale community health checks for older adults. The effectiveness of PAPLICA has also been verified, and it has reported good sensitivity, specificity, positive preditcitve value and negative predictive value [12].

Although cognitive domains decline with age, their decline is not uniform [13, 14]. For example, memory and processing speed decline with age, whereas language related to vocabulary and language comprehension remain constant throughout life [15–19]. Furthermore, visuospatial function declines rapidly after reaching a specific age [20]. Considering that PAPLICA can measure multiple cognitive domains, we expect that the influence of aging varies by domain, such as language and visuospatial functions, and it is significantly affected by aging beyond a certain age. By contrast, attention, memory, and abstract thinking gradually decrease with age, thus resulting in a linear decline in test scores.

Although we conducted various health checkups using PAPLICA, the influence of aging and years of education on cognitive function has not yet been examined. Therefore, this study aimed (1) to determine whether PAPLICA can detect the influencesof aging and years of education and (2) to propose an appropriate administration of the tests.

We hypothesize that the results obtained by PAPLICA will be similar to those obtained by other neuropsychological tests (i.e., the number of years of education is correlated with higher scores, and higher age is correlated with lower test scores).

## Methods

### Participants

We recruited participants for a health promotion event via the local government and explained to participants in advance, both verbally and in writing, that there would be health checkups as part of the program. Twelve local governments recruited participants for health promotion events in their districts. We established a priori inclusion and exclusion criteria for this study. The inclusion criteria were age 60 years or older and the ability to come to the event site alone. The exclusion criteria were participants with a medical and psychiatric disorder and dementia. We used the participants’ self-reports of psychiatric disorder and dementia to determine whether participants met the exclusion criteria.

### Measures

The primary outcome was the score for each test included in PAPLICA. We also collected demographic data on the participants’ age, sex, and years of education.

### Instruments

The examiner presented the task to the participants by using a personal computer and a projector. The participants wrote their answers using a pencil provided in the booklet.

#### PAPLICA

PAPLICA measures five cognitive functions (attention, memory, language, visuospatial, and abstract thinking) using 10 tests. PAPLICA requires approximately 25 min to perform but can be performed with approximately 15 participants at a time by 1 examiner and 1 assistant, if necessary, without special equipment (only a projector).

PAPLICA consists of 10 tests. Speed I and Speed II were used to measure attention, the Word Delayed Recall Test (Word DRT) and Picture Enhanced Cued Recall Test (Picture ECR) (including free recall) were used to evaluate memory, CLOX I and CLOX II were used to assess visuospatial function, the Letter Fluency Test (LFT) and Category Fluency Test (CFT) were used to measure language, and the Smilarity was used to evaluate abstract thinking.

PAPLICA has a unique advantage compared to conventional assessments such as MMSE and MoCA, as it can test multiple participants at once instead of conducting individual tests for each participant. This enables screening more participants, which was impossible with previous assessments.

#### Procedure

**Fig. 1:**
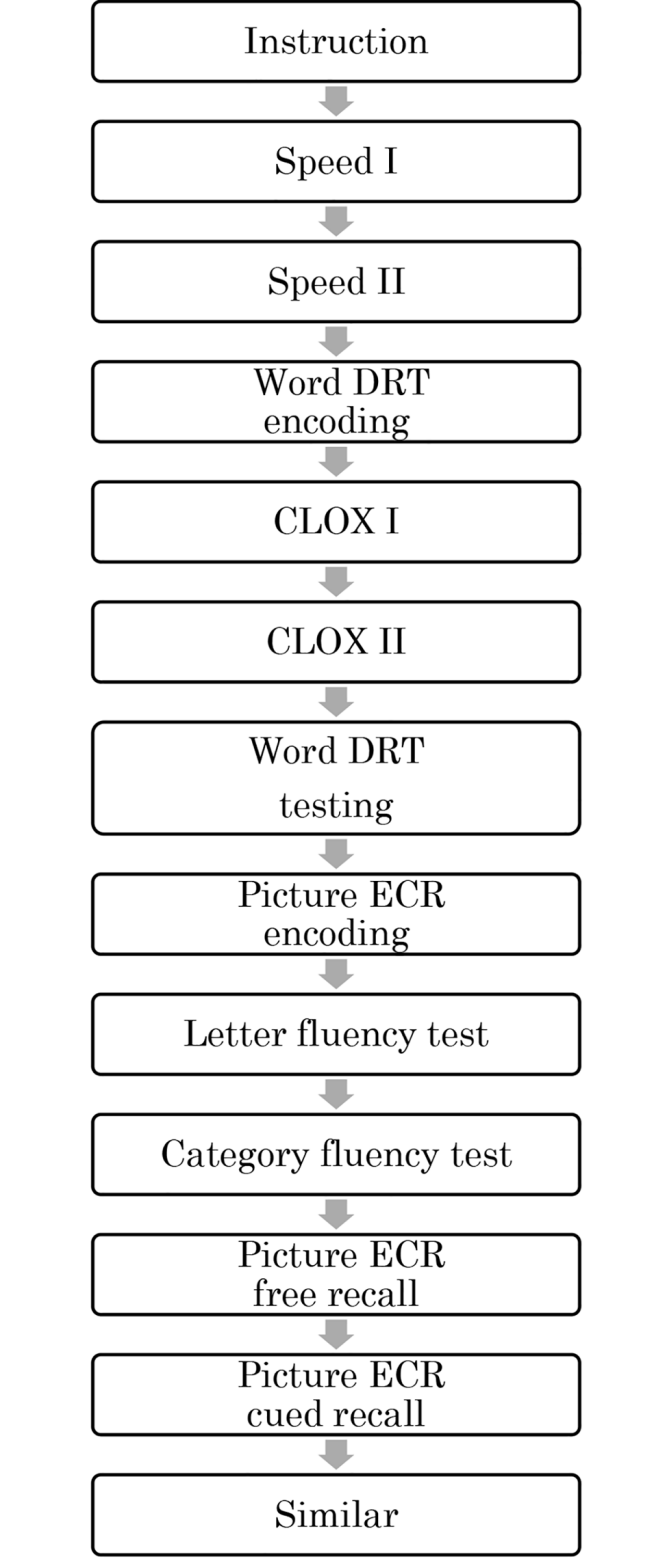
Procedure flowchart of PAPLICA

One examiner performed the test with approximately 15 participants. The examiner performed the tests in the order shown in Fig. 1. First, the examiner distributed the booklet to the participants and instructed them not to turn to the next page and not to discuss the answer with other participants.　Then, the examiner asked the participants to checkups whether they could see the screen and hear the instructions. Once all participants confirmed that they were able to see the screen and hear the instructions, the test began.

Psychomotor function may affect their scores. Therefore, psychomotor function was measured using Speed I and was entered as a covariate.

### Speed

Speed I and Speed II were used. Speed I test sheets were printed blank in 11 circles across five rows. The examiner asked the participants to write as many numbers as possible in empty circles within 30 s.

In Speed II, participants wrote numbers and Hiragana characters alternately in empty circles, with numbers in order and Hiragana characters following theorder of the Japanese syllabary. Speed I test scores included the number of correctly completed items, whereas Speed II scores included the number of items completed in alternating numerals and Hiragana, with digits in order and Hiragana in syllabic order.

### Word DRT (encoding)

The Word DRT was administered in two parts (encoding and testing). Participants were asked to memorize five words that were read twice by the examiner. After the last word, the examiner told the participants to recall the words they had learned later.

### CLOX

CLOX consists of CLOX I and CLOX II. In CLOX I, the participants drew a clock according to the following instructions: outline the clock, distribute numbers on the face, and draw a needle pointed at 1:45.

In CLOX II, the participants copied the clock projected onto the screen within two minutes. The CLOX score was scored by two independent scorers, excluding the examiner, by using identical scoring criteria.

### Word DRT (test)

In the test part, the examiner asked participants to recall as many words as possible and write them down on the sheet within 60 s regardless of the typography or order in which the examiner read aloud. The test score was the sum of the words that the participant wrote correctly.

### Picture ECR (encoding)

The Picture ECR was administered in two parts (encoding and testing). The examiner asked the participants to memorize the names of eight pictures. The examiner called out four item names and category names while the participants looked at the card. The eight pictures were presented in two separate sessions (four at a time), and the participants were told to recall them later.

### Word fluency test

The word fluency test consists of an LFT and a CFT. Participants remembered many words beginning with “TA” in this task and wrote them down in any order. The test was conducted for 60 s. Following the LFT, the CFT followed the same procedure as the LFT, except that words belonging to the animal category were recalled and written down by participants. The LFT score is the sum of words beginning with the letter specified by the instruction.

The CFT score is the sum of words in the categories specified by the instruction. Words other than the specified letter, those outside the specified category, and those repeated more than once were excluded.

### Picture ECR (test)

Immediately after the WFT, the Picture ECR test was administered. In the first test, participants recalled eight items that the examiner had read at the encoding stage and wrote them down as many as possible regardless of order and typography. In the next test, the participants engaged in a cue replay test using eight category names. Both tests were performed for 60 s. Each score was the number of correct responses that the participant gave to the eight words presented in the previous encoding.

### Similarity

First, as an example, the examiner read two words (carrot and burdock), and the features common to the two words were represented (vegetable). Thereafter, the participant responded to the features common to each of the 8-word pairs in the booklet within 180 s. Two scorers scored the responses independently, and the total number of matched responses was used as the abstract thinking score.

### Statistical analysis

To analyze the years of education, we used independent sample *t*-tests to assess the test scores of the 2 groups (≤ 12 years and > 12 years of education). To analyze aging, we used analysis of covariance to assess the 5 age groups (60–69, 70–74, 75–79, 80–84, and 85≤ ), with years of education and Speed I test scores as covariates. Multiple comparisons were performed using the Bonferroni correction. The significance level for all analyses was set at 0.05, with the exception of multiple comparisons. The Cohen d and ${\eta }_{p}^{2}$ were used to determine effect sizes. SPSS version 23 (IBM Corp., Armonk, NY, USA) was used to perform the statistical analyses.

## Results

### Study population

**Fig. 2:**
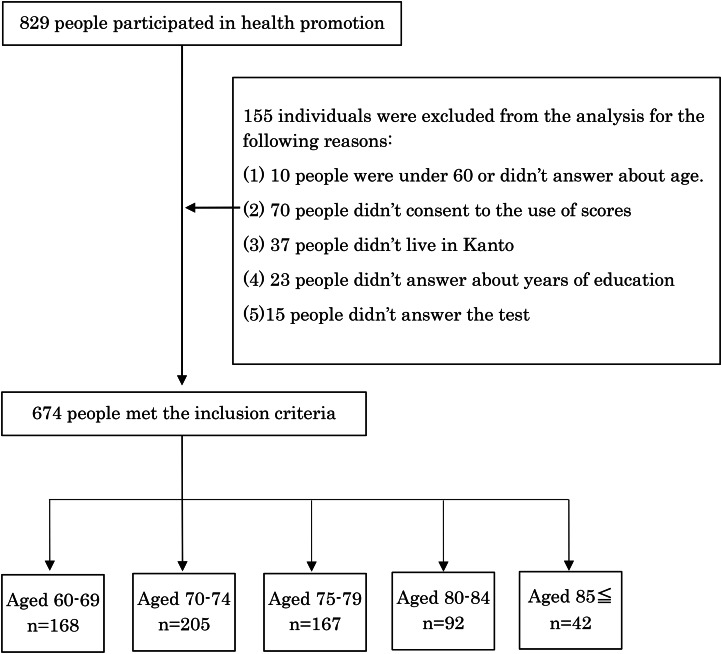
CONSORT DIAGRAM of this study

In total, 829 participants participated in the health promotion program, but 674 individuals were included in the analysis (Fig. 2).

### Years of education

To determine the influence of years of education on cognitive function, we used 12 years as the criterion, with ≤ 12 years as the low education group (*n* = 372, Age_median_ = 75, SD = 6.15) and > 12 years as the high education group (*n* = 302, Age_median_ = 72, SD = 5.69). In the analysis, except for CLOX I scores, the scores of the higher education group were higher than those of the lower education group (*ps* < 0.05). These results indicate that, in addition to years of education affecting cognitive function, PAPLICA adequately detects the influence of years of education.

### Influence of aging

Demographic data for each of age groups are shown in Table 1. Table 2 shows the mean and standard error of the test scores by age group. The analysis showed that, except for Speed I (*F* [4, 667] = 1.94, *p* = 0.103, ${\eta }_{p}^{2}$ = 0.011) and LFT scores (*F* [4, 667] = 1.05, *p* = 0.381, ${\eta }_{p}^{2}$ = 0.006), the cognitive test scores declined with age (*p* < 0.001). However, the age at which the influence of aging became more pronounced differed among tests (Table 2).

**Table 1:** Demographic data of participants by age group (*n* = 674)

Group	60–69 n = 168	70–74 n = 205	75–79 n = 167	80–84 n = 92	85≦ n = 42	*p* -value^2^	Effect size ${\eta }_{p}^{2}$
Females, % (n)	84% (141)	88% (181)	90% (150)	60% (55)	74% (31)	*-*	-
Age,^1^ years (SD)	67(1.75) _a_	72 (1.45) _b_	77(1.46) _c_	81 (1.23) _d_	86 (1.66) _e_	*p* < 0.000	0.234
Years of Education^1^ (SD)	14(1.99) _a_	13 (2.46) _a_	12 (2.48) _b_	12 (2.87) _b_	10.5(3.04) _c_	*p* < 0.000	0.002

Notes: ^1^ The numbers entered in the Age and Years of Education rows represent the median. ^2^ The significant level of ANOVA was set at 0.05. Alphabets appended to the values entered in the Age and Years of Education rows indicate statistical differences between conditions

Abbreviations: SD, standard deviation

**Table 2:** Descriptive statistics by cognitive function tests and results of ANCOVA^1,2^ and Post hoc comparisons (*n* = 674)

Test	Score range	60–69 (n = 168)	70–74 (n = 205)	75–79 (n = 167)	80–84 (n = 92)	85≦ (n = 42)	*p* -value ^3^	Effect size ${\eta }_{p}^{2}$
		M (SE)	M (SE)	M (SE)	M (SE)	M (SE)		
Speed I	0 ~ ∞	33.61 (0.43)	32.28 (0.39)	29.96 (0.44)	28.20 (0.68)	25.71 (1.27)	*p* = 0.103	0.011
Speed II	0 ~ ∞	25.70 (0.42) _a_	22.82 (0.39) _b_	20.43 (0.50) _b_	19.83 (0.62) _b_	16.24 (0.97) _c_	*p* < 0.001	0.076
CLOX I	0 ~ 10	9.55 (0.06) _a_	9.24 (0.07) _a_	9.20 (0.10) _a_	9.09 (0.11) _a_	8.60 (0.31) _b_	*p* < 0.001	0.029
CLOX II	0 ~ 10	9.84 (0.03) _a_	9.78 (0.04) _a_	9.75 (0.04) _a_	9.70 (0.06) _a_	9.31 (0.14) _b_	*p* < 0.001	0.034
Word DRT	0 ~ 5	4.32 (0.07) _a_	4.09 (0.06) _a_	3.62 (0.11) _b_	3.73 (0.12) _c_	2.33 (0.27) _d_	*p* < 0.001	0.098
Picture ECR Free recall	0 ~ 8	6.36 (0.11) _a_	5.67 (0.11) _b_	5.08 (0.16) _b_	5.32 (0.18) _a_	3.88 (0.43) _c_	*p* < 0.001	0.060
Picture ECR Cued recall	0 ~ 8	7.64 (0.08) _a_	7.38 (0.07) _a_	6.85 (0.14) _b_	6.87 (0.18) _a_	5.55 (0.46) _c_	*p* < 0.001	0.058
LFT	0 ~ ∞	8.77 (0.19)	8.47 (0.18)	7.98 (0.20)	7.74 (0.31)	6.69 (0.44)	*p* = 0.381	0.006
CFT	0 ~ ∞	13.49 (0.24) _a_	13.00 (0.23) _a_	12.03 (0.24) _a_	10.58 (0.38) _b_	9.52 (0.51) _b_	*p* < 0.001	0.047
Similarity	0 ~ 8	6.20 (0.10) _a_	5.87 (0.10) _a_	5.19(0.14) _b_	5.01(0.20) _b_	3.95 (0.30) _c_	*p* < 0.001	0.055

Abbreviations: SE, standard error; DRT, delayed recall test ECR, enhanced cued recall; LFT, letter fluency test; CFT, category fluency test

Notes: ^1^ The number of years of education and the number of Speed I responses were entered as covariates. ^2^ Multiple comparisons were

performed using the Bonferroni method as a posteriori test. ^3^ The significance level for ANCOVA was 0.05 and 0.001 for multiple comparisons

using Bonferroni. Different alphabets assigned to scores within the same row indicate a significant difference

## Discussion

In this study, we examined whether PAPLICA could detect the effects of aging and years of education. The results confirmed the influence of aging and years of education on cognitive function.

### Detection of the influence of years of education and age

PAPLICA detected the influence of years of education and age on cognitive decline. The number of years of education is a risk factor for cognitive decline, and more years of education are associated with less dementia [1, 21]. In the present analysis of the years of education, all scores of the participants with > 12 years, except for the CLOX I scores, were higher than those with ≤ 12 years. Therefore, we conclude that PAPLICA could detect the influence of years of education on cognitive function in the same way as other tests.

Previous studies have reported age-related cognitive decline [19]. In the current study, each test score declined with age. Furthermore, the pattern of decline differed by cognitive domain. The influence of aging on each cognitive domain was discussed in terms of fluid and crystallized intelligence, with cognitive functions derived from fluid intelligence declining with age. By contrast, cognitive functions derived from crystallized intelligence are maintained throughout life [22–24]. Regarding the classification of cognitive functions in terms of intelligence, processing speed, attention, memory, and executive functions were considered fluid intelligence; language was considered crystallized intelligence; and visual space was considered fluid and crystalline intelligences [25]. The results of the present study replicate the results obtained by standardized tests.Thus, PAPLICA can detect cognitive decline even a in large-scale health checkup and enable early intervention of the examinee. It is expected that this test will contribute to the health of more people in the future.

### Strengths

In addition to detecting the effects of aging and years of education, PAPLICA and other tests have the advantage of evaluating more participants at one time than the MMSE or MoCA tests. Even if the scale of health checkups is large, the tests can be conducted at multiple sites, thus making it possible to guide participants without waiting. Additionally, a short inspection time is expected to minimize the impact of participant fatigue on the test results.

The PAPLICA used in this study included tests in which scores decline with aging and neurodegeneration. Therefore, it is possible to make quantitative judgments regarding the scores obtained from the tests and qualitative judgments regarding whether the decline in scores is due to aging or neurodegeneration. It is also possible to obtain a multifaceted view of the state of the participants’ cognitive function, and the results can provide more participants’ information than ever before.

### Limitations and future scope

This study used a large dataset, and the results were in agreement with those obtained from other tests, thus indicating that PAPLICA can detect the effects of aging. However, the validity of the following points should be noted. In this study, neurologic disorders, dementia, and MCI were excluded based on self-reporting, thus indicating the possibility that a certain number of participants may have been included. it is first necessary to study participants with age-appropriate cognitive function who do not have a neurological disease or MCI to establish a standard value that can detect an early decline in cognitive function. In addition, comparing our results of people with dementia to those with MCI may help clarify the background of cognitive decline by sorting out the dissimilarities with the pattern of decline due to aging. The study involved healthy seniors living in the community. However, in large-scale health check-ups, there is a possibility that seniors with limitations in reading and writing, as well as those with visual or hearing impairments, may participate. Therefore, it is necessary to consider implementation methods that allow seniors with diverse backgrounds to participate in health check-ups.Furthermore, as the present study did not compare PAPLICA with cognitive test batteries, it is necessary to demonstrate the usefulness of PAPLICA by comaring the sensitibity and specificity of other cognitive test batteries.

The examiner’s behavior can also be considered a future task. In PAPLICA, individual interventions may influence other participants. When conducted by trained professionals, they can observe participants’ behavior during the assessment and provide interventions considering other participants. As a result, more individuals are likely to demonstrate their cognitive abilities during the assessment. Therefore, developing training programs for examiners is also an important future task.

## Conclusions

Our newly developed PAPLICA can detect the effects of educational history and aging and can be performed in a short time and in groups. Although this study demonstrates the usefulness of PAPLICA, there are limitations to consider. By examining the aforementioned issues, the test will be able to detect early decline in cognitive function, even in large-scale community health check-ups.

## Data Availability

Data supporting the findings of this study are available from the corresponding author upon reasonable request.

## Abbreviations

PAPLICA: Paper–pencil type group examination for cognitive assessment
MCI: Mild cognitive impairment
MMSE: Mini-Mental State Examination
MoCA: Montreal Cognitive Assessment
TMIG: Tokyo Metropolitan Institute of Gerontology
Word DRT: Word Delayed Recall Test
Picture ECR: Picture Enhanced Cued Recall Test
LFT: Letter fluency test
CFT: Category fluency test
SD: Standard deviation
SE: Standard error
DRT: Delayed recall test
ECT: Enhanced cued recall test

## References

[1] Livingston G, Sommerlad A, Orgeta V, Costafreda SG, Huntley J, Ames D (2017). Dementia prevention, intervention, and care: 2020 report of the Lancet Commission. Lancet.

[2] Cabinet Office. : Heisei 29th edition of the White Book on Ageing Society (Outline Edition). [in Japanese] https://www8.cao.go.jp/kourei/whitepaper/w-2017/html/gaiyou/s1_2_3.html. Accessed 8 June 2023.

[3] Stern Y, Arenaza-Urquijo E, Bartrés-Faz D, Belleville S, Cantilon M, Chetelat G (2020). Whitepaper: defining and investigating cognitive reserve, brain reserve, and brain maintenance. Alzheimers Dement.

[4] Iizuka A, Suzuki H, Ogawa S, Takahashi T, Cho D, Yamashiro D (2021). Randomized controlled trial of the picture book reading program on cognitive function in middle-aged people. Front Psychiatry.

[5] Iizuka A, Suzuki H, Ogawa S, Kobayashi-Cuya KE, Kobayashi M, Inagaki H (2019). Does social interaction influence the effect of cognitive intervention program? A randomized controlled trial using Go game. Int J Geriatr Psychiatry.

[6] Park DC, Lodi-Smith J, Drew L, Haber S, Hebrank A, Bischof GN (2014). The impact of sustained engagement on cognitive function in older adults: the Synapse Project. Psychol Sci.

[7] Verghese J, LeValley A, Derby C, Kuslansky G, Katz M, Hall C (2006). Leisure activities and the risk of amnestic mild cognitive impairment in the older adults. Neurology.

[8] Suzuki H, Kuraoka M, Yasunaga M, Nonaka K, Sakurai R, Takeuchi R (2014). Cognitive intervention through a training program for picture book reading in community-dwelling older adults: a randomized controlled trial. BMC Geriatr.

[9] Canevelli M, Grande G, Lacorte E, Quarchioni E, Cesari M, Mariani C (2016). Spontaneous reversion of mild cognitive impairment to normal cognition: a systematic review of literature and meta-analysis. J Am Med Dir Assoc.

[10] Folstein MF, Folstein SE, McHugh PR (1975). Mini-mental state”. A practical method for grading the cognitive state of patients for the clinician. J Psychiatr Res.

[11] Nasreddine ZS, Phillips NA, Bédirian V, Charbonneau S, Whitehead V, Collin I (2005). The Montreal Cognitive Assessment, MoCA: a brief screening tool for mild cognitive impairment. J Am Geriatr Soc.

[12] Suzuki H, (principale Investigator). Development of the group version of the cognitive assessment test using a video (Project No. 22790583). MEXT KAKENHI.2010–2011. https://kaken.nii.ac.jp/ja/file/KAKENHI-PROJECT-22790583/22790583seika.pdf. Accessed 8 June 2023.

[13] Hartshorne JK, Germine LT (2015). When does cognitive functioning peak? The asynchronous rise and fall of different cognitive abilities across the life span. Psychol Sci.

[14] Tashiro D, Nakahara M, Tanaka K, Murooka M, Haraguchi K (2019). Comparison of cognitive functions based on MMSE/MoCA-J scores among different age groups of older adults community residents. Rigakuryoho Kagaku.

[15] Lindenberger U, Baltes PB (1997). Intellectual functioning in old and very old age: cross-sectional results from the Berlin Aging Study. Psychol Aging.

[16] Rönnlund M, Nyberg L, Bäckman L, Nilsson LG (2005). Stability, growth, and decline in adult life span development of declarative memory: cross-sectional and longitudinal data from a population-based study. Psychol Aging.

[17] Salthouse TA (2004). What and when of cognitive aging. Curr Dir Psychol Sci.

[18] Salthouse TA (2010). Selective review of cognitive aging. J Int Neuropsychol Soc.

[19] Schaie KW. Developmental influences on adult intelligence: the Seattle Longitudinal Study. 2nd ed. Oxford University Press; 2013.

[20] Konagaya Y, Watanabe T, Konagaya M (2012). Cognitive function screening of community-dwelling older adults people using the clock drawing test -quantitative and qualitative analyses. Nippon Ronen Igakkai Zasshi.

[21] Stern Y, Gurland B, Tatemichi TK, Tang MX, Wilder D, Mayeux R (1994). Influence of education and occupation on the incidence of Alzheimer’s disease. JAMA.

[22] Park DC, Lautenschlager G, Hedden T, Davidson NS, Smith AD, Smith PK (2002). Models of visuospatial and verbal memory across the adult life span. Psychol Aging.

[23] Park DC, Reuter-Lorenz P (2009). The adaptive brain: aging and neurocognitive scaffolding. Annu Rev Psychol.

[24] Salthouse T (2012). Consequences of age-related cognitive declines. Annu Rev Psychol.

[25] Harada CN, Natelson Love MCN, Triebel KL (2013). Normal cognitive aging. Clin Geriatr Med.

